# The Evaluation of Prognostic Value and Immune Characteristics of Ferroptosis-Related Genes in Lung Squamous Cell Carcinoma

**DOI:** 10.1055/s-0043-1776386

**Published:** 2023-10-30

**Authors:** Jialin Su, Shuhua Tan, Houwu Gong, Yongzhong Luo, Tianli Cheng, Hua Yang, Xiaoping Wen, Zhou Jiang, Yuning Li, Lemeng Zhang

**Affiliations:** 1 Thoracic Medicine Department 1, Hunan Cancer Hospital, Changsha, Hunan Province, People's Republic of China; 2 School of Life and Health Sciences, Hunan University of Science and Technology, Xiangtan, People's Republic of China; 3 College of Computer Science and Electronic Engineering, Hunan University, Changsha, People's Republic of China

**Keywords:** lung squamous carcinoma, ferroptosis, prognostic model, immune infiltration, antitumor immunity

## Abstract

**Background** The purpose of our study was to construct a prognostic model based on ferroptosis-related gene signature to improve the prognosis prediction of lung squamous carcinoma (LUSC).

**Methods** The mRNA expression profiles and clinical data of LUSC patients were downloaded. LUSC-related essential differentially expressed genes were integrated for further analysis. Prognostic gene signatures were identified through random forest regression and univariate Cox regression analyses for constructing a prognostic model. Finally, in a preliminary experiment, we used the reverse transcription-quantitative polymerase chain reaction assay to verify the relationship between the expression of three prognostic gene features and ferroptosis.

**Results** Fifty-six ferroptosis-related essential genes were identified by using integrated analysis. Among these, three prognostic gene signatures (HELLS, POLR2H, and POLE2) were identified, which were positively affected by LUSC prognosis but negatively affected by immune cell infiltration. Significant overexpression of immune checkpoint genes occurred in the high-risk group. In preliminary experiments, we confirmed that the occurrence of ferroptosis can reduce three prognostic gene signature expression.

**Conclusions** The three ferroptosis-related genes could predict the LUSC prognostic risk of antitumor immunity.

## Introduction

Lung squamous carcinoma (LUSC) arises from morphological changes in the bronchial epithelium during chronic inflammation and is characterized by accelerated and aggressive progression. 1 LUSC accounts for 30% of nonsmall cell lung cancer (NSCLC) histological types, with unique mutational and genomic profiles. 2 More than 400,000 patients with LUSC are newly diagnosed each year. 3 Because of specific clinicopathological features, including venerable age, delayed diagnosis, comorbid diseases, and tumor centrality, the median survival of patients with LUSC is approximately 30% shorter than that of other NSCLC subtypes, 4 whereas the 5-year survival rate for patients with NSCLC is only 15%. 5 Immunomodulatory therapy is an effective treatment for tumor recurrence and metastasis by disrupting inhibitory signaling between tumor and immune cells. 6 However, even with interventional immunotherapy, the treatment and prognosis of LUSC remain challenging. Therefore, it is of great significance to explore LUSC-specific prognostic features to predict immunotherapy response, prevent tumor recurrence, and improve clinical outcomes for patients with LUSC.

Ferroptosis is used to describe cell death characterized by iron overload, accumulation of lipid reactive oxygen species, and lipid peroxidation. 7 In contrast to programmed death, such as necrosis, apoptosis, and autophagy, ferroptosis has unique morphological characteristics, biological functions, and transcriptional regulation mechanisms. 8 Ferroptosis-related physiological differences also exist between cancer and normal cells; therefore, ferroptosis plays a key role in inhibiting tumorigenesis by removing cells in the tumor microenvironment that are deficient in nutrients or damaged by stress. 9 Induction of ferroptosis has become a viable replacement therapy to activate the death of tumor cells, especially for malignant tumors that are resistant to conventional therapies. 10 Studies have investigated the ferroptosis-related pathways that may inhibit NSCLC and found that the expression of several key oncogenes may be associated with ferroptosis escape and resistance. 11 12 Relevant studies have also reported that some ferroptosis-related genes can be used to predict prognostic survival in laryngeal, oral, and head and neck squamous cell carcinoma. 13 14 15 Additionally, overexpressed p63 in LUSC promotes tumorigenesis through ferroptosis-related oxidative stress responses. 16 However, there is a lack of comprehensive and systematic studies on whether ferroptosis-related genes affect LUSC prognosis.

Benefitting from the comprehensive development of sequencing technology and the shared gene atlas, this study used bioinformatics methods to integrate the expression profile data from the Cancer Genome Atlas (TCGA) and Gene Expression Omnibus (GEO) databases and to screen more reliable ferroptosis-related LUSC essential genes. Using random forest regression and univariate Cox regression analyses, gene signatures significantly associated with LUSC prognosis were obtained, followed by the construction of prognostic models. We also explored the potential relationships between gene signatures, the immune microenvironment, and mutation profiles. Our findings suggest three prognostic biomarkers associated with ferroptosis and offer the possibility of improving the outcome of patients with LUSC.

## Materials and Methods

### Data Preparation and Processing

The RNA-seq data and clinical information of 493 LUSC samples and 49 adjacent normal samples were collected from TCGA. 17 Besides, the gene expression data of GSE19188, containing 27 LUSC tumor samples and 65 normal samples, were downloaded from the GEO database based on GPL570 platform ([HG-U133_Plus_2] Affymetrix Human Genome U133 Plus 2.0 Array). For each dataset, the Ensembl_ID was converted to the corresponding gene symbol according to the gene annotation file in the Gencode database (hg38, V22). 18 When multiple Ensembl_ IDs were mapped to the same gene symbol, the average expression level was used. These two datasets were used to identify ferroptosis-related gene signatures. Moreover, the expression data of GSE126044 consisting 5 anti-PD-1 responders and 11 nonresponders were downloaded from GEO database (GPL16791 platform) to predict the immunotherapy effects of the identified signatures.

### Screening of DEGs between Lung Squamous Carcinoma and Normal Samples

For data from GSE19188, the differentially expressed genes (DEGs) between LUSC and normal samples were determined by using the *t* -test in R package “Limma” (version 3.10.3), 19 and genes with |log fold change (FC)| > 1 and adjusted *p* -value < 0.05 were considered as DEGs. Similarly, in terms of TCGA RNA-seq data, we used Limma package to screen DEGs between LUSC and normal samples. The |logFC| > 1 and adjusted *p* -value < 0.05 were set as thresholds.

### Identifying the Essential Lung Squamous Carcinoma Genes-Related to Ferroptosis

To screen the essential genes of LUSC, the genome-wide CRISPR data of LUSC cells were downloaded from DepMap ( https://depmap.org/portal/download/ ). 20 The dependence scores of approximately 17,000 candidate genes were calculated using the CERES algorithm. Then, candidate genes with CERES score < − 1 and accounting for more than 75% of all samples were defined as essential genes. Subsequently, these essential genes were intersected with two groups of up-regulated and down-regulated DEGs (TCGA and GSE19188), and the overlapping genes with logFC > 1 were regarded as up-regulated essential genes in tumor tissues, 21 which were displayed in a Venn diagram.

Further, the ferroptosis-related genes were obtained from the FerrDb database ( http://www.zhounan.org/ferrdb/ ), 22 which included drivers (genes that drive ferroptosis), suppressors (genes that inhibit ferroptosis), and markers (genes that indicate ferroptosis). The R.cor function ( http://77.66.12.57/R-help/cor.test.html ) was applied to calculate the Pearson correlation coefficient (PCC) between essential genes and ferroptosis-related genes. Genes with |PCC| > 0.05 and *p* -value < 0.05 were considered as ferroptosis-related essential genes.

### Functional Enrichment Analyses of Ferroptosis-Related Essential Genes

To observe the biological functions of ferroptosis-related essential genes, the GO-BP terms and KEGG pathways analyses were performed using clusterProfiler 3.16.0 (version: 3.16.0, http://bioconductor.org/packages/release/bioc/html/clusterProfiler.html ). 23 Statistical significance was set at a gene count ≥ 2 and *p* -value < 0.05.

### Random Forest Regression Analysis

Based on the ferroptosis-related essential genes, the random forest model was constructed to rank the genes with their significance using R3.6.1 randomForest (version: 4.6-14, https://www.stat.berkeley.edu/~breiman/RandomForests/ ). 24 Then, the top 30 genes were selected as the key ferroptosis-related essential genes of LUSC.

### Screening of Prognosis-Related Genes

To evaluate the prognostic value of key genes, all TCGA-LUSC samples were grouped into a training set (259 cases) and validation set (198 cases) at a ratio of 3:2. In the training set, the univariate Cox regression analysis was performed to investigate prognostic-related key genes using the Survival package (version 3.2-7, http://bioconductor.org/packages/survival/ ). 25 Genes with *p* -value less than 0.05 were identified as gene signatures.

### Construction and Validation of Prognostic Model

Further, multivariate Cox analysis of gene significances was conducted to construct prognostic model using the survival R package. The risk score for each patient was calculated based on the following formula:

Risk score = ∑Coef _gene_ ×Exp _gene_ .

Of which, Coef indicates the coefficient of gene calculated in the multivariate Cox regression analysis, and Exp indicates the gene expression levels.

To assess the performance of the constructed prognostic model, patients in the TCGA training set, validation set, and total set were divided into high- and low-risk groups according to the medium risk score. Further, KM curves were generated to compare the OS rates of the low-risk and high-risk groups. Moreover, samples were grouped into high- and low-expression based on the optimal cut-off value of each gene signature. KM analysis was employed to assess the prognostic difference between two groups. *p* -Value < 0.05 was considered statistically significant.

### Independent Analysis of the Prognostic Model

Univariate and multivariate Cox analyses were used to explore whether the predictive power of prognostic model could be independent of other clinical characteristics (including age, gender, stage, pathologic T, pathologic M, and pathologic N stage) of patients with LUSC using log-tank test. *p* -Value < 0.05 indicated statistical significance.

### Correlation Analysis of Immune Infiltration and Gene Expression

The TIMER online tool ( https://cistrome.shinyapps.io/timer/ ) 26 was used to explore the correlation between the expression level of gene signatures and the degree of immune cell infiltration in LUSC samples.

### Verification of Protein Expression Levels

The human protein atlas (HPA) online database ( https://www.proteinatlas.org/ ) is a valuable tool for researchers to study the spatial map of the human proteome, which records the distribution and expression level of each protein in 48 normal human tissues, 20 tumor tissues, 47 cell lines, and 21 blood cells. 27 In this study, we used HPA database to search the immunohistochemistry images and protein expression levels of gene signatures in the prognostic model.

### Assessment of the Anti-PD-1 Therapy Response

We retrieved a dataset (GSE126044) from GEO database that studies the different responses of patients after PD-1 immunotherapy for lung cancer. The expression data of gene signatures in the prognostic model were extracted from GSE126044 and then compared the expression difference between anti-PD-1 responders and nonresponders.

### Association Analysis of High/Low-Risk Groups and Immune Microenvironment

To explore the potential tumor immunotherapy effect of gene signatures, the expression level of immune checkpoints, including *PDCD1* , *CD274* , *CTLA4* , *ICOS* , *HAVCR2* , *LAG3* , *CD73* , *CD47* , *BTLA* , *TIGIT* , *SIRPA* , *TNFRSF4* , *TNFRSF9* , and *VTCN1* , was extracted and then compared their expression differences between high- and low-risk groups. Moreover, we also observed the relationship between the different risk groups and immune cell fractions. In brief, the Cibersort algorithm 28 was applied to analyze the infiltration of 22 immune cell types (set parameter: perm = 100, QN = F) in the LUSC samples, and the difference in the infiltration abundance between the high- and low-risk groups was compared. *p* -Value < 0.05 was considered statistically significant.

### Screening of DEGs between High-Risk and Low-Risk Groups

DEGs between high-risk and low-risk groups were screened by R.limma package. DEGs were selected with the threshold of |logFC| > 2 and *p* -value < 0.05, followed by functional enrichment analysis.

### Tumor Mutation Burden Analysis

The somatic mutation data of LUSC were downloaded from TCGA using software MuTect. Tumor mutation burden (TMB) between low-risk and high-risk groups was analyzed by Maftools (version 2.0.16, https://github.com/PoisonAlien/maftools ) 29 and visualized in summary plot.

### Cell Culture

Human lung squamous cell lines SK-MES-1, HCI-H520, and HCI-H226 and normal lung epithelial cell lines BEAS2B were purchased from Changsha Visier Biotechnology Co., Ltd, and frozen in liquid nitrogen. The cells were cultured in rpm-1640 medium (Hyclone, Logan, UT) containing 10% fetal bovine serum (FBS; Gibco, Grand Island, NY, United States), with 100 U/mL penicillin and 100 µg/mL streptomycin (Hyclone). The cells were stored in a humidified incubator at 37°C with 5% CO2.

### Inducing Ferroptosis and Detecting Fe2+

Intracellular ferrous iron (Fe2 + ) level was determined using the iron assay kit (Beijing Solarbio Science & Technology Co., Ltd., China) according to the manufacturer's instructions. Cells were seeded onto a 10 cm ^2^ plate (5 × 106 cells per plate) and treated with RSL3 (KKL Med Inc. Ashland, VA, United States) or dimethyl sulfoxide for 24 hours. Cells were collected and washed in ice-cold phosphate-buffered saline and homogenized in 5× volumes of iron assay buffer on ice and, then, centrifuged (13,000 × g, 10 minutes) at 4°C to remove insoluble material. After discarding the supernatant, each sample was mixed with an iron reducer before being incubated for 30 minutes at room temperature. After the addition of 100 µL of the iron probe to each sample, the reactivity was mixed together and permitted to run at room temperature in the dark for an hour. A colorimetric microplate reader has been employed to take an immediately apparent readout of the absorbance at 593 nm.

Reverse Transcription-Quantitative Polymerase Chain Reaction Total RNA was extracted from lung cancer cells using RNA Cell/Tissue Mini Kit (Ecotop Scientific Co., China). A reverse transcription kit (Toroivd Technology Co., China) was used to reverse transcribe RNA, and mRNA expression was evaluated using the 2(ΔΔCt) method. Relative gene expression was then calculated and normalized to endogenous glyceraldehyde 3-phosphate dehydrogenase (GAPDH). The primers of GAPDH,HELLS,POLE2,POLR2H were purchased from Sangon Biotech (Shanghai) Co., Ltd. China. The forward and reverse primer sequences are as follows: GAPDH F: 5′-ACAGCCTCAAGATCATCAGC-3′ and GAPDH R: 5′-GGTCATGAGTCCTTCCACGAT-3′, HELLS F: 5′-CCCTCCTTTCTTCTAGTAATGCAGTT-3′ and HELLS R:

5′-CCCAATCTCTCCCCATGAAAA-3′;POLE2 F: 5′-CCCAGATATTCACCAAAGTAGTCG-3′ and POLE2 R:5′-TGGTGGCCTTGGTAAGATGG-3′;POLR2H: 5′-CGTACTCCCAACTGTGGTCG-3′ and POLR2H R:5′-TCGGTCAAACTTCTTGCCCT-3′.

### Statistical Analysis

The visualized using GraphPad Prism 8 was used for analysis. Using a two-tailed *t* -test to examine the data, significant differences were defined as *p* < 0.05. The data were expressed as mean ± standard error. All experiments were repeated thrice.

## Results

### Screening of Ferroptosis-Related Essential Genes in Lung Squamous Carcinoma

**Fig. 1:**
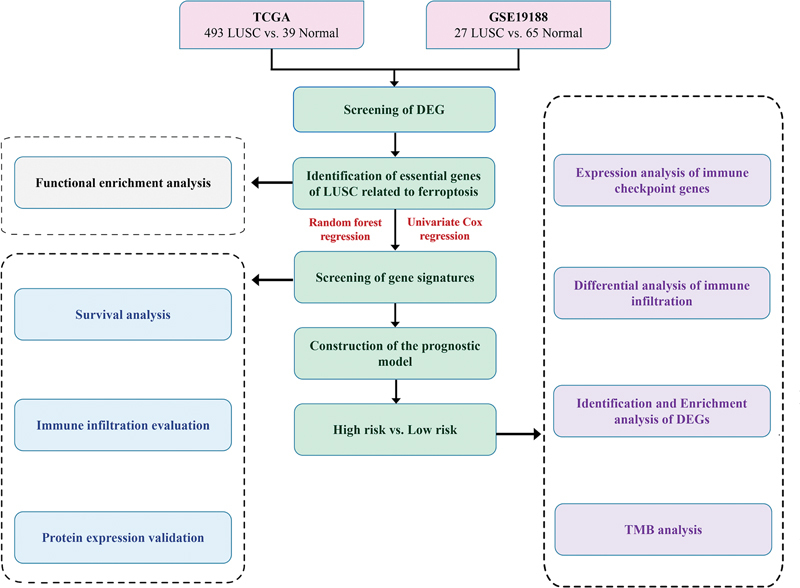
Workflow of this study.

**Fig. 2:**
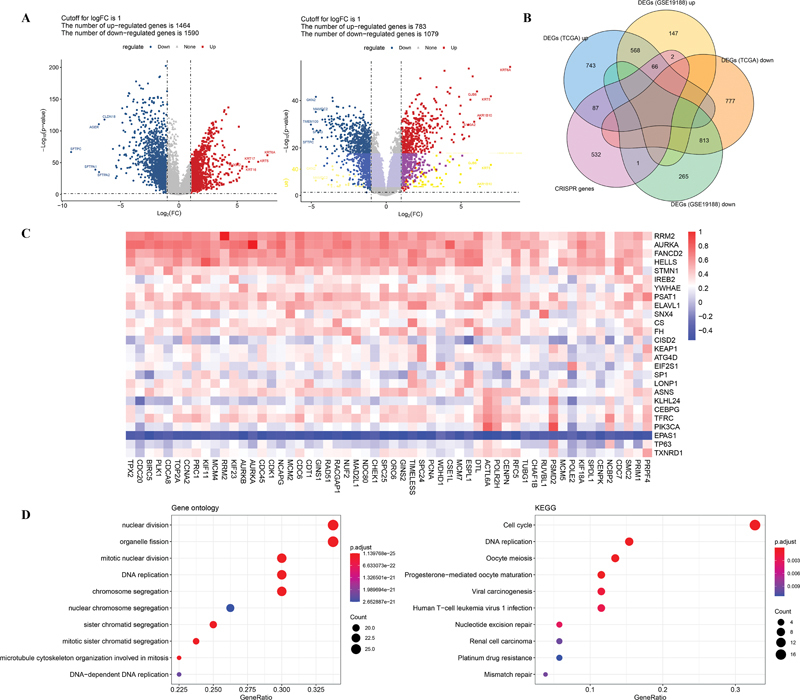
Screening of ferroptosis-related essential genes for LUSC. ( **A** ) Volcano plots showing the DEGs between LUSC and normal samples from TCGA ( *left* ) and GSE19188 ( *right* ) datasets. ( **B** ) Identification of essential genes of LUSC: Venn diagram of *TCGA* , *GSE19188* , and *GRISPR* genes. ( **C** ) Correlation analysis between LUSC essential genes and ferroptosis-related genes at the expression level. ( **D** ) The enriched GO-BP terms ( *left* ) and KEGG pathways ( *right* ) of 56 ferroptosis-related essential genes.

The data sources and workflow of this study are summarized in Fig. 1 . After differential analysis, 3,054 DEGs (1464 up-regulated and 1590 down-regulated) from TCGA and 1,862 DEGs (783 up-regulated and 1,079 down-regulated) from GSE19188 were obtained between LUSC and normal samples ( Fig. 2A ). A total of 688 essential genes of LUSC were screened using DepMap database. Next, the Venn diagram was used to screen for common genes among three databases (DepMap, up-regulated DEGs in TCGA and GSE19188), and 66 up-regulated essential genes were identified for further analysis ( Fig. 2B ). Next, the correlation between the 66 essential genes and ferroptosis-related genes was evaluated. As shown in Fig. 2C , 176 coexpression pairs, involving 56 essential genes and 26 ferroptosis-related genes, were obtained. Thus, these 56 genes were defined as ferroptosis-related essential genes. The expression level of these genes in TCGA and GSE19188 is shown in Table 1 .

**Table 1:** Information of 56 essential genes of LUSC associated with ferroptosis

Essential genes	TCGA		GSE19188	
	logFC	Adjusted *p* -value	logFC	Adjusted *p* -value
TPX2	3.91199812	2.50E–133	3.648108803	1.27E–34
CDC20	3.875582979	1.03E–130	3.719428265	4.26E–36
BIRC5	3.693534074	1.53E–117	2.378543869	1.59E–39
PLK1	2.820323637	1.42E–112	1.482443436	9.13E–29
CDCA8	2.932718732	8.56E–110	1.93194843	4.62E–33
TOP2A	3.642976708	3.52E–104	3.053562294	2.68E–32
CCNA2	2.973667777	4.78E–104	2.396803926	1.69E–30
PRC1	2.813146737	7.56E–104	3.283098831	5.97E–33
KIF11	2.546216881	4.03E–102	2.569138695	6.43E–27
MCM4	2.678564461	1.90E–101	1.573963595	3.33E–38
RRM2	3.217014522	3.11E–100	3.687855235	7.34E–25
KIF23	2.298509853	3.20E–97	2.124227513	8.70E–29
AURKB	3.079879928	4.98E–97	1.398943156	3.45E–31
AURKA	2.518594888	5.01E–97	1.867053963	2.40E–33
CDC45	2.972506655	1.38E–95	2.414235219	3.66E–34
CDK1	2.785567048	1.13E–93	2.216631911	7.70E–30
NCAPG	2.097406446	1.36E–89	2.429003317	9.58E–33
MCM2	2.95973815	1.96E–89	2.472629389	3.14E–34
CDC6	2.602130936	2.68E–89	2.139892516	5.78E–33
CDT1	2.591314125	1.57E–88	1.611906833	1.12E–26
GINS1	2.512413352	3.72E–88	3.584296514	4.35E–37
RAD51	2.099814854	5.31E–85	1.194037589	5.03E–33
RACGAP1	2.041523867	6.93E–85	2.130435132	1.94E–28
NUF2	2.555935138	2.38E–81	3.378421755	4.63E–37
MAD2L1	2.220105124	5.80E–78	2.055071076	2.40E–28
NDC80	2.268048132	6.53E–78	3.104784286	3.27E–27
CHEK1	1.728838353	3.05E–77	1.503435875	1.12E–30
SPC25	2.04172006	6.38E–74	2.256038388	6.98E–28
ORC6	1.971258788	6.81E–74	2.110752126	3.48E–31
GINS2	2.39354602	1.57E–73	1.083216209	2.37E–32
TIMELESS	1.994293003	5.65E–72	1.06156867	8.46E–32
SPC24	2.131459057	1.45E–71	1.341409405	5.17E–29
PCNA	1.891587069	1.72E–66	1.417010347	2.68E–22
WDHD1	1.535671251	8.39E–66	1.070067727	4.81E–29
CSE1L	1.504466098	5.11E–65	1.013329558	3.39E–29
MCM7	1.982682575	1.92E–63	1.492195555	2.45E–28
ESPL1	1.718318801	2.12E–63	1.386111055	2.84E–29
DTL	1.938140335	3.10E–62	2.685959093	6.59E–24
ACTL6A	2.169920969	8.58E–60	1.819222578	5.14E–31
POLR2H	1.870437082	5.92E–56	1.622909527	4.62E–31
CENPN	1.60853151	8.66E–56	1.269691635	1.53E–27
RFC5	1.493308377	3.83E–55	1.285685437	2.06E–20
TUBG1	1.357005463	3.33E–54	1.200892794	4.92E–19
CHAF1B	1.501626962	8.45E–54	1.02764823	6.68E–22
RUVBL1	1.434809921	6.47E–52	1.112601418	6.69E–17
PSMD2	1.700377801	1.53E–49	1.46480786	3.19E–32
MCM5	1.496992995	1.76E–48	1.199106977	2.59E–18
POLE2	1.420213439	9.66E–48	1.802714305	5.34E–29
KIF18A	1.280002678	9.00E–46	1.347054986	3.28E–20
SPDL1	1.018223246	4.41E–45	1.332911496	2.59E–19
CENPK	1.24032718	1.35E–43	1.869429106	9.65E–20
NCBP2	1.328220898	7.32E–43	1.317916114	9.56E–27
CDC7	1.202797018	3.43E–34	1.065141378	3.39E–10
SMC2	1.132903029	2.07E–29	1.367094402	6.61E–20
PRIM1	1.069432193	9.29E–28	1.235039803	2.87E–18
PRPF4	1.00483375	1.08E–25	1.033975023	1.35E–16

Abbreviations: FC, fold change; LUSC, lung squamous carcinoma; TCGA, the cancer genome atlas.

*p* -Value was adjusted by the Benjamini and Hochberg method.

To further explore the biological function of these genes, Gene Ontology-biological processes (GO-BPs) and Kyoto Encyclopedia of Genes and Genomes (KEGG) pathways analyses were performed. Results revealed that these genes were significantly enriched in 432 GO-BP terms and 20 KEGG pathways. Among them, the top 10 GO-BP terms and pathways are shown in Fig. 2D . The results suggested that these ferroptosis-related essential genes were mainly enriched in nuclear division and organelle fission (GO-BP term) and cell cycle (KEGG pathway).

### Screening of Prognostic Gene Signatures of Lung Squamous Carcinoma

**Fig. 3:**
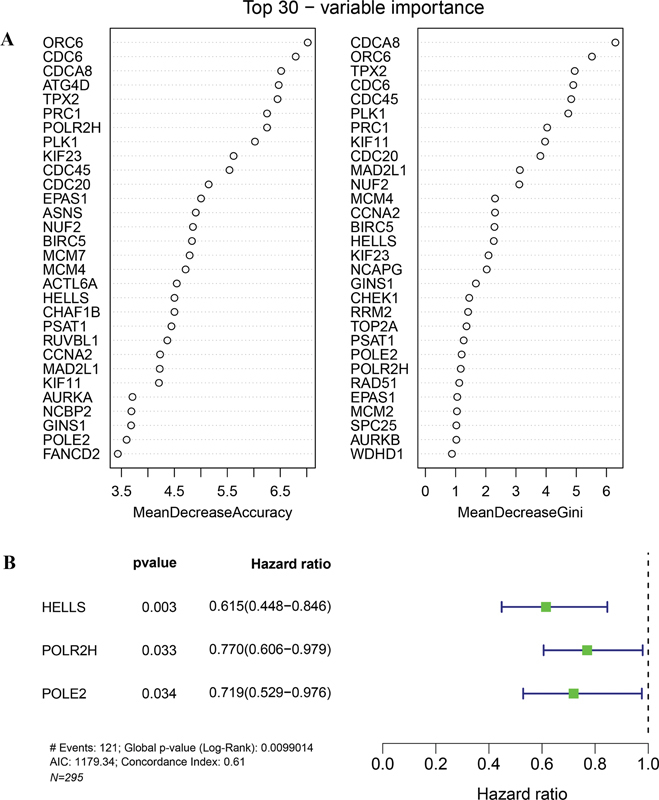
Identification and construction of gene signatures significantly associated with LUSC prognosis. ( **A** ) Top 30 genes for predicting survival according to mean decrease and Gini index evaluated by random forest regression analysis. ( **B** ) Forest map of three gene signatures ( *HELLS* , *POLR2H* , and *POLE2* ) of LUSC prognosis that identified using univariate Cox regression analysis.

A random forest model was constructed to rank the importance of ferroptosis-related essential genes. The mean decrease in Gini measure was computed to assess the importance of variables. Greater significance was assigned to variables with a higher mean decline than to those with a lower mean decrease, which were identified as key genes. The top 30 key genes are presented in Fig. 3A , including *CDCA8* , *ORC6* , *TPX2* , and *CDC6* .

Next, in the training set, univariate and multivariate analyses were conducted, and three genes, including *HELLS* , *POLR2H* , and *POLE2* , were finally selected to construct the prognostic model ( Fig. 3B ). The C-index of the risk score was 0.61, suggesting that the constructed model had a superior prognostic value. The risk score was calculated using the following formula: risk score = (Exp _*HELLS*_ * − 0.357415015) + (Exp _*POLR2H*_ * − 0.135790324) + (Exp _*POLE2*_ * − 0.144240097).

### Construction and Verification of the Prognostic Model

**Fig. 4:**
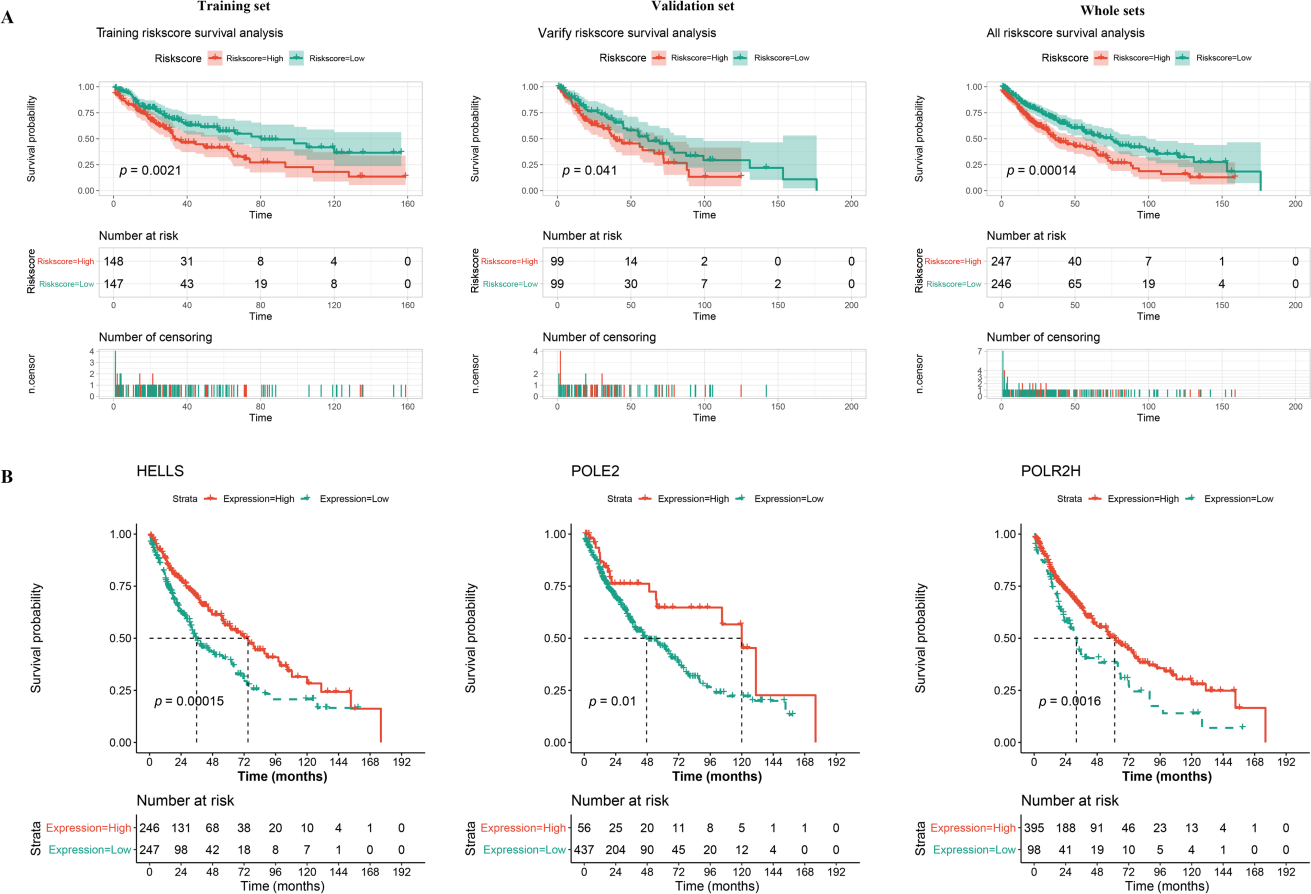
Validation of the prognostic model. ( **A** ) Kaplan–Meier curve of the three-gene signature in the training set, validation set, and whole set. ( **B** ) Prognostic role of *HELLS* , *POLR2H* , and *POLE2* were evaluated by using Kaplan–Meier analysis.

Based on the calculated median risk score, patients in the training set, validation set, and total set were divided into high- and low-risk groups. Kaplan–Meier (KM) suggested that patients in all three datasets with high-risk scores had significantly poorer survival probability than those with low-risk scores ( Fig. 4A , all *p* < 0.05). Next, survival analysis of three genes in the prognostic model was also performed. The result showed that patients with higher expression of *HELLS* , *POLR2H* , and *POLE2* had remarkably better survival status than those with low expression ( Fig. 4B , *p* < 0.05), indicating that the three gene signatures may play roles as protectors in the prognosis of LUSC.

### Evaluation of Independent Prognostic Value of Risk Score

Univariate and multivariate Cox regression analyses were performed to evaluate the prognostic model as an independent prognostic factor for LUSC. Univariate analysis showed that risk score, stage, pathologic T, and pathologic M were considered to have prognostic values. Further, multivariate analysis showed that risk score was independently associated with prognostic of LUSC patients ( Table 2 , *p* < 0.05).

**Table 2:** Identification of independent prognostic factors using the univariate and multivariate Cox regression analyses

Clinical characteristics	Univariable Cox		Multivariable Cox	
	HR (95%CI)	*p* -Value	HR (95%CI)	*p* -Value
Riskscore	1.702 (1.292–2.243)	0 a	1.851 (1.368–2.506)	0 a
Pathologic_T	1.341 (1.124–1.601)	0.001 a	1.167 (0.915–1.487)	0.214
Stage	1.277 (1.082–1.507)	0.004 a	1.111 (0.871–1.418)	0.396
Pathologic_M	3.088 (1.261–7.557)	0.014 a	2.062 (0.752–5.655)	0.16
Pathologic_N	1.146 (0.942–1.394)	0.174		
Age	1.282 (0.865–1.899)	0.215		
Gender	1.193 (0.866–1.645)	0.28		

Abbreviations: CI, confidence interval; HR, hazard ratio.

a *p* -Value < 0.05 indicates statistical significance.

### Association between Gene Signatures and Immune Infiltration

**Fig. 5:**
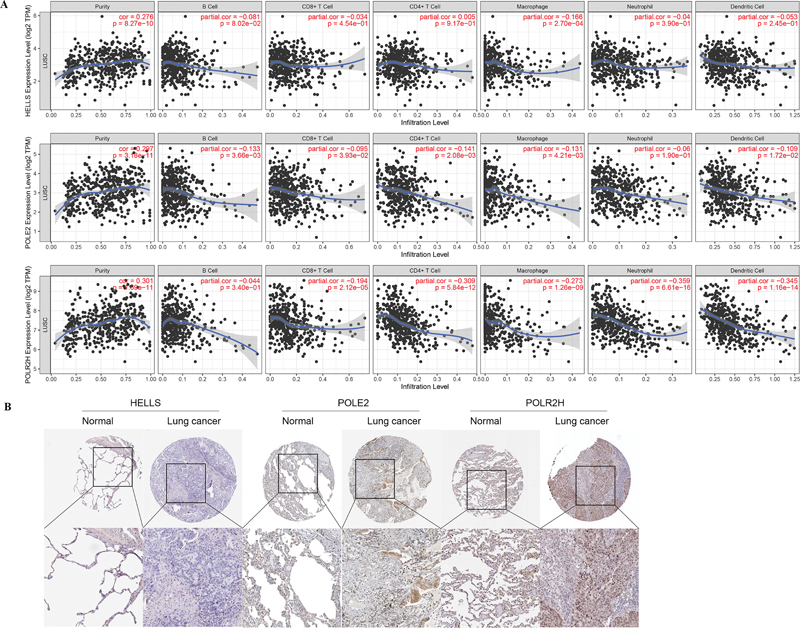
Immunoinfiltration analysis and protein immunohistochemical validation. ( **A** ) Correlation analysis between immune cells and gene expression of three gene signature. ( **B** ) Immunohistochemistry of *HELLS* , *POLR2H* , and *POLE2* in lung cancer and normal tissues based on the Human Protein Atlas database.

The TIMER online tool was used to analyze the association between three gene signatures and immune infiltration. As shown in Fig. 5A , the *HELLS* expression level was significantly negatively associated with macrophages; the *POLE2* expression level was significantly negatively correlated with the infiltration of B cells, CD8+ T cells, CD4+ T cells, macrophages, and dendritic cells; and the *POLR2H* expression level was negatively related to T cell, macrophage, neutrophil, and dendritic cell infiltration. Moreover, it was also determined that the expression of *HELLS* , *POLR2H* , and *POLE2* was significantly positively associated with tumor purity in LUSC.

### Validation of Protein Expression Level of Gene Signatures

Next, we also explored the protein expression levels of three gene signatures using HPA database. The immunohistochemical results showed that the three gene signatures had high expression characteristics in lung cancer compared with those in normal tissues ( Fig. 5B ).

### Prediction of Potential Treatment Response to Anti-PD-1 Drug

To predict the potential response to anti-PD-1 drug, the expression levels of *HELLS* , *POLR2H* , and *POLE2* were extracted from the GSE126044 dataset. However, no correlation was found between three gene expression level and anti-PD-1 therapy responses in patients with NSCLC, indicating that these genes are not predictive of immunotherapy responses ( Supplementary Fig. S1 , available in the online version).

### Expression of Immune Checkpoint Genes between Different Risk Groups

**Fig. 6:**
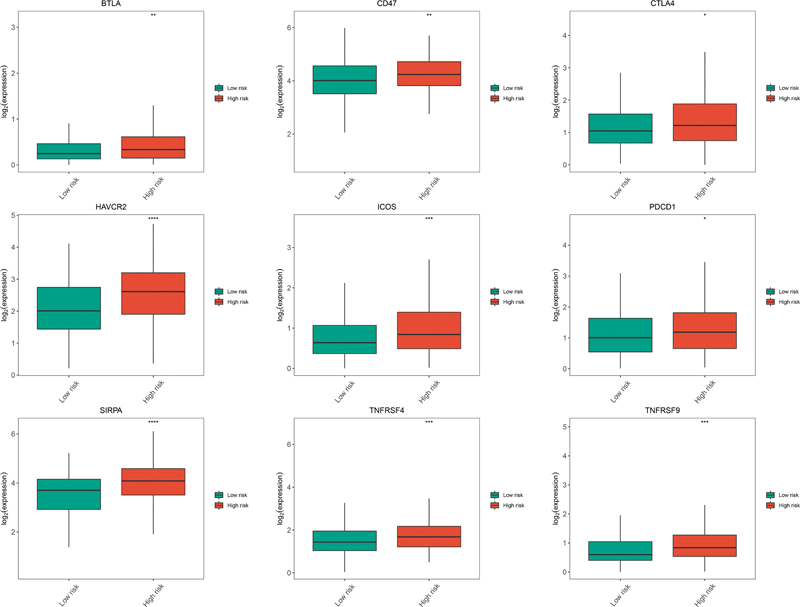
Expression differences of immune checkpoint genes ( *BTLA* , *CD47* , *CTLA4* , *HAVCR2* , *ICOS* , *PDCD1* , *SIRPA* , *TNFRSF4* , and *TNFRSF9* ) between high-risk and low-risk groups. * *p* < 0.05; ** *p* < 0.01; *** *p* < 0.001. Green indicates low-risk group and red indicates high-risk group.

To evaluate the potential effects of gene signatures on immunotherapy, we detected differences in the expression of several immune checkpoints between high- and low-risk groups. The results showed that *BTLA* , *CD47* , *CTLA4* , *HAVCR2* , *ICOS* , *PDCD1* , *SIRPA* , *TNFRSF4* , and *TNFRSF9* were significantly overexpressed in the high-risk group ( Fig. 6 , *p* < 0.05). This further suggested that the poor prognosis in patients with LUSC at high prognostic risk could be caused by their inertia in antitumor immunity.

### Difference in Immune Infiltration between Risk Groups

**Fig. 7:**
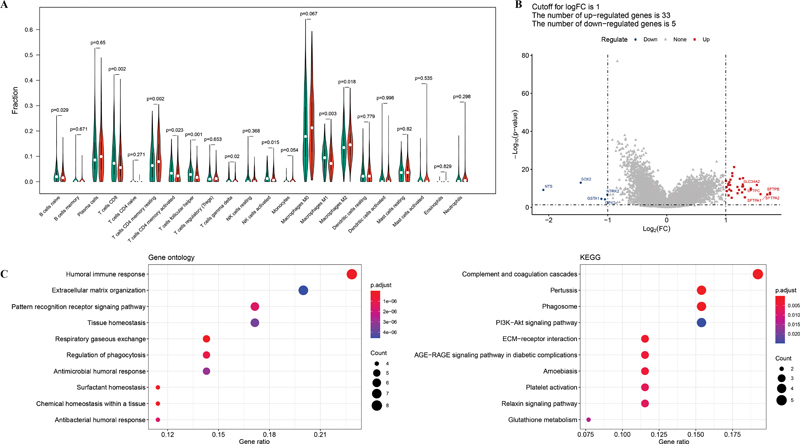
Differences in immune infiltration and gene expression between high-risk and low-risk groups were compared. ( **A** ) The difference of the immune infiltration in different risk groups. The red bar indicates the high-risk group and the green bar indicates the low-risk group. *p* < 0.05 was considered statistically significant. ( **B** ) Volcano plot showing DEGs between high- and low-risk groups. Blue node indicates down-regulated gene and red node indicates up-regulated gene. ( **C** ) Enriched GO terms ( *left* ) and KEGG pathways ( *right* ) of 38 DEGs between the high- and low-risk groups.

The difference in immune cell infiltration between patients in the high- and low-risk groups was analyzed. The results suggested that nine immune cells, including naïve B cells, CD8 T cells, resting memory CD4 T cells, activated memory CD4 T cells, follicular T helper cells, γδT cells, activated NK cells, M1 macrophages, and M2 macrophages, showed significant differences in infiltration abundance between the two groups ( Fig. 7A ).

### Screening of DEGs between Low-Risk and High-Risk Groups

Moreover, 38 DEGs between low-risk and high-risk groups were screened, including 33 up-regulated and 5 down-regulated DEGs ( Fig. 7B ). Functional enrichment analyses revealed that these DEGs were significantly involved in 184 GO functions and 16 KEGG pathways. For example, these DEGs were mainly enriched in BP for humoral immune response and KEGG pathways for complement and coagulation cascades ( Fig. 7C ).

### Analysis of Tumor Mutation Burden between Two Risk Groups

**Fig. 8:**
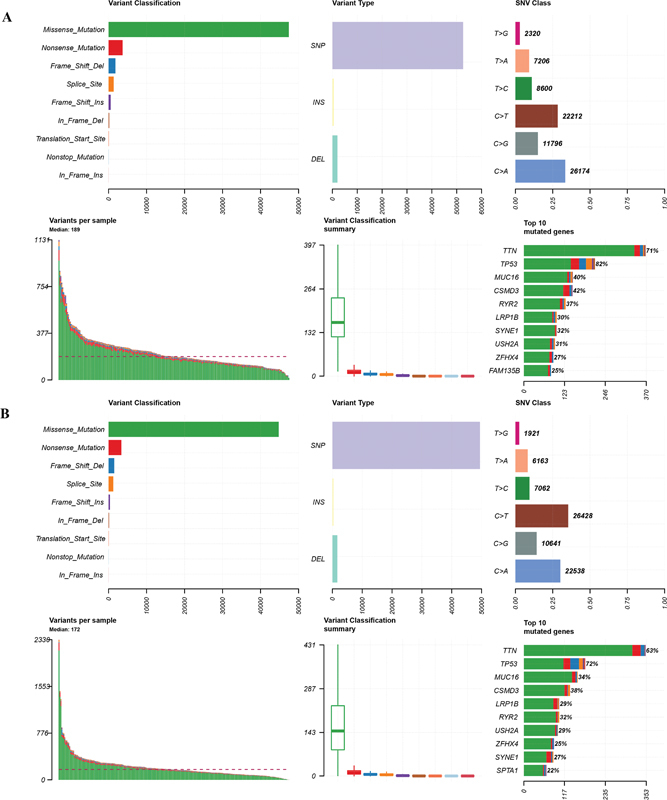
Summary plots show the tumor mutational burden of ( **A** ) low-risk and ( **B** ) high-risk groups in terms of variant classification, variant type, SNV class, variant per sample, and top 10 mutated genes.

Finally, the TMB of each risk group was analyzed, and summary plots were created accordingly. As shown in Fig. 8 , the majority of variants were missense mutations, whereas single nucleotide polymorphisms were the main variant type in the two groups. The top 10 genes with the highest mutation frequency in patients with low prognostic risk included *TTN* , *TP53* , *MUC16* , *CSMD3* , *RYR2* , *LRP1B* , *SYNE1* , *USH2A* , *ZFHX4* , and *FAM135B* ( Fig. 8A ). The top 10 genes distributed in patients at high prognostic risk were *TTN* , *TP53* , *MUC16* , *CSMD3* , *LRP1B* , *RYR2* , *USH2A* , *ZFHX4* , *SYNE1* , and *SPTA1* ( Fig. 8B ). Furthermore, we found lower *TTN* and *TP53* mutations in the high-risk group than in the low-risk group, indicating a relationship between benign *TTN/TP53* mutations and favorable LUSC prognosis.

### The Gene Signatures are Highly Expressed in Human Lung Squamous Cell Lines

**Fig. 9:**
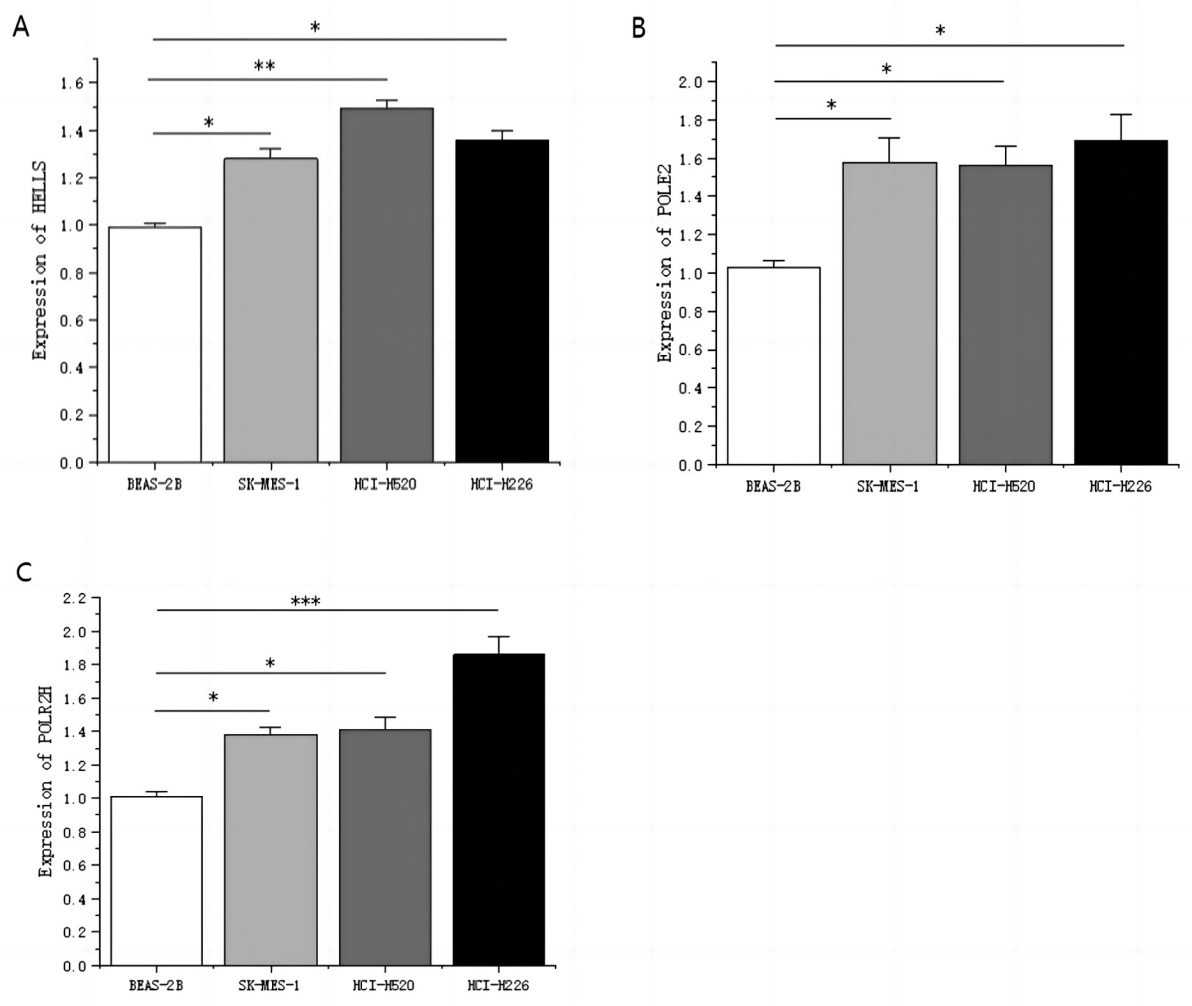
( **A** – **C** ) Expression levels of HELLS, POLE2, and POLR2H in squamous cell lines and normal lung epithelial cells.* *p* < 0.05; ** *p* < 0.01; *** *p* < 0.001.

To further evaluate the expression of gene signatures, we measured mRNA levels in lung squamous cell lines and normal lung epithelial cells using the reverse transcription-quantitative polymerase chain reaction (RT-qPCR). When compared with the normal lung epithelial cell BEAS2B, genes HELLS ( Fig. 9A ), POLE2 ( Fig. 9B ), and POLR2H ( Fig. 9C ) mRNA levels in human lung squamous cell lines SK-MES-1, HCI-H520, and HCI-H226 were remarkably highly expressed.

### Ferroptosis Reduced the Expression Level of Gene Signatures

**Fig. 10:**
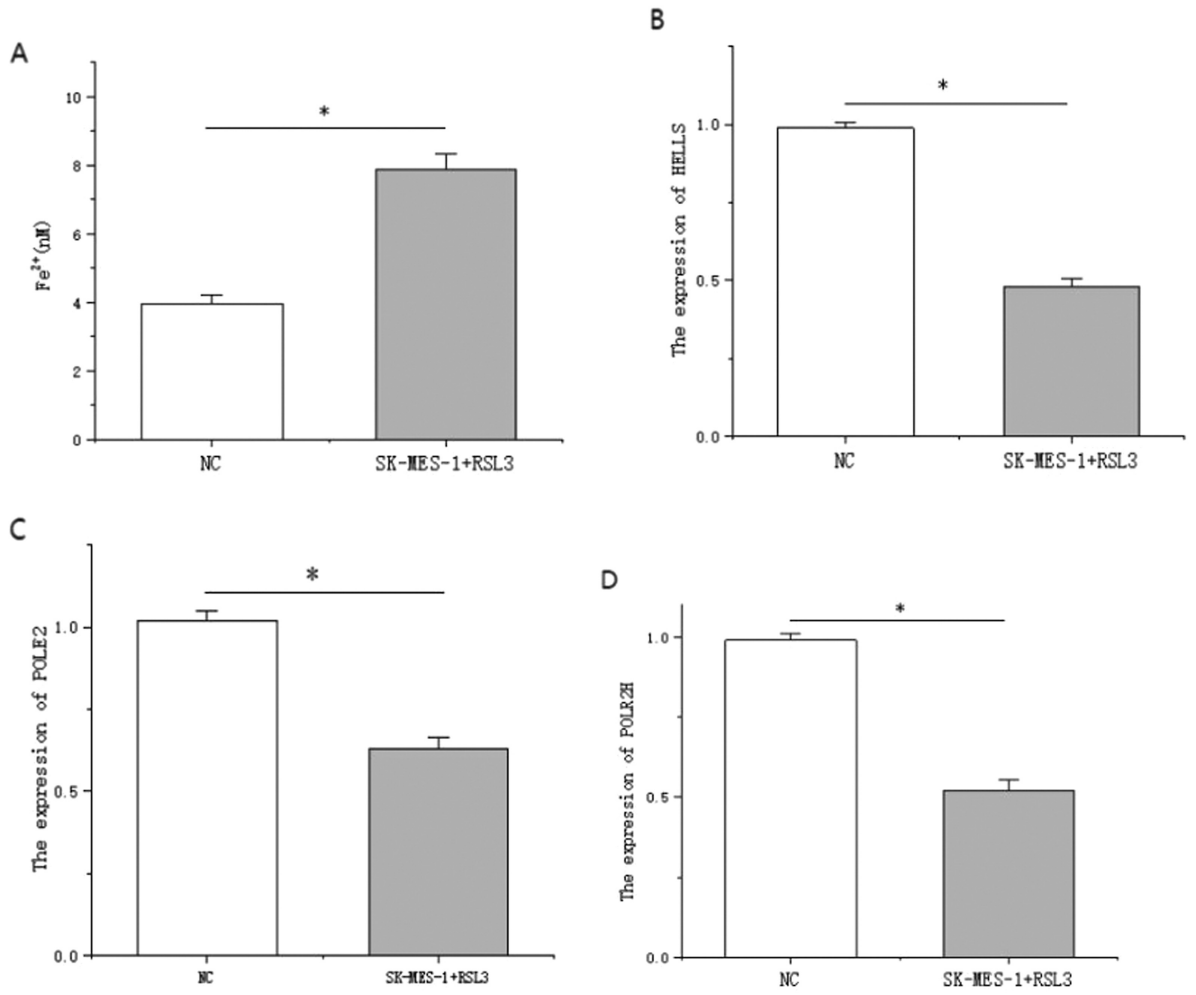
( **A** ) The Fe ^2+^ level of lung squamous cell SK-MES-1 changed after adding RSL3. ( **B** – **D** ) The expression levels of HELLS, POLE2, and POLR2H in SK-MES-1 cells were changed after ferroptosis induced by RSL3. * *p* < 0.05.

To verify the occurrence of iron death and the expression of genes HELLS, POLE2, and POLR2H, we used iron death inducer RSL3 to intervene SK-MES-1 lung squamous cancer cells. Iron death in lung squamous cell SK-MES-1 was determined by detecting a significant increase in Fe2+ content of SK-MES-1 using the iron assay kit ( Fig. 10A ). Using RT-qPCR, we found that the expression of target genes HELLS, POLE2, and POLR2H in the experimental group was significantly down-regulated compared with the control group ( Fig. 10B–D ).

## Discussion

The unfavorable prognosis of LUSC is associated with the lack of effective targeted therapies because of its unique clinicopathological and molecular features. 30 Therefore, the identification of LUSC-specific prognostic biomarkers is important for the development of effective targeted therapies for patients with LUSC. Moreover, ferroptosis is an iron- and reactive oxygen species-dependent cell death, which is mainly characterized by cytological changes, such as reduction or disappearance of mitochondrial cristae, and mitochondrial membrane condensation. 31 Notably, these cell abnormalities are due to the occurrence of strong membrane lipid peroxidation and oxidative stress that further lead to the loss of selective permeability of the plasma membrane. Previous evidence has indicated that many types of squamous cell carcinoma are sensitive to ferroptosis, and ferroptosis-related genes act as independent prognostic predictors for other subtypes of NSCLC. 32 33 For example, Diao et al 34 identified a set of 16 ferroptosis-related genes (e.g., *CAV1* , *ATF3* , *HELLS* , *PLIN2* , and *TFRC* ) to develop a gene signature that could accurately predict the prognosis of LUSC, suggesting that targeting these ferroptosis-related genes may serve as a novel therapeutic alternative for LUSC. In addition, key regulators in ferroptosis including *SLC7A11* , *GPX4* , and *AIFM2* were dysregulated in a variety of tumors (such as LUSC) and were candidate prognostic biomarkers for many types of cancer and could be used to assess the infiltration of immune cells in immune cells in tumor tissues. 35 Nevertheless, few studies have reported the ferroptosis-related genes that are associated with the prognosis for LUSC patients.

In this study, we screened 56 LUSC essential genes that correlated with ferroptosis drivers, suppressors, and markers. These genes were mainly enriched in the functions of nuclear division, organelle fission, and the cell cycle pathway. In combination with prognostic information from LUSC samples, we identified three gene signatures ( *HELLS* , *POLR2H* , and *POLE2* ) as protective factors in LUSC samples using regression analyses. The expression of these genes was negatively correlated with the infiltration of major immune cells but significantly positively correlated with tumor purity. Risk score-based prognostic models were constructed based on three gene signatures to independently predict LUSC prognosis risks. Additionally, we also found differences in immune cell infiltration and immune checkpoint gene expression between high-risk and low-risk samples, indicating the potential role of the three gene signatures in antitumor immunity.

In this study, three potential biomarkers of LUSC ( *HELLS* , *POLR2H* , and *POLE2* ) were selected from ferroptosis-related genes that were significantly upregulated in LUSC. Among them, *HELLS* silencing can inhibit the proliferation of small cell lung cancer in vitro by regulating mTOR and apoptotic signaling pathways. 36 Although *HELLS* was significantly upregulated in LUSC in this study, it was not sufficient to account for its tumor-promoting effect. Yano et al believed that *HELLS* is a tumor-suppressor gene of NSCLC located on chromosome 10. 37 The survival analysis in our study suggested that patients with LUSC with high *HELLS* expression had a better prognosis. Zhu et al supported our conclusion and demonstrated that the improved prognosis for lung cancer was related to an increase in the mRNA expression of *HELLS* . 38 Furthermore, the expression of *POLE2* is believed to inhibit the proliferation and apoptosis of A549 and NCI-H1299 cells. 39 Another protector of LUSC prognosis risk, *POLR2H* , is also associated with unclassified lung cancer survival. 40 These findings partly explain the positive role of gene signatures in LUSC prognosis, which does not contradict their high expression in LUSC samples because our findings also propose a significant negative correlation between their expression and infiltration of major immune cells. Evidence has shown that the malignant phenotype of LUSC is often accompanied by the recruitment of infiltrated immune cells in the tumor immune microenvironment. 41 Therefore, we hypothesized that *HELLS* , *POLR2H* , and *POLE2* may inhibit the malignant proliferation of tumor cells by slowing the invasion of immune cells.

As an immunosuppressive malignant tumor, the malignant phenotype of LUSC is influenced by the tumor microenvironment along with the high infiltration of multiple immune cells, such as macrophages, neutrophils, dendritic cells, B cells, and T cells. 42 In this study, based on the risk score classification, the LUSC prognostic risk groups differed significantly in the infiltration abundance of B cells, T cells, macrophages, and dendritic cells. Among them, the expression of the B cell translocation gene *BTG1* had high sensitivity and specificity for predicting the prognosis of NSCLC. 43 Moreover, T cell receptor repertoires and the T cell-related gene *TIM* - *3* could potentially be used to predict the prognosis of patients with NSCLC. 44 With regard to macrophages, multifactorial analysis of the related study illustrated that a high proportion of M2 macrophages was significantly associated with poor OS status of NSCLC. 45 Our results also suggested a significantly higher M2 macrophage infiltration abundance in patients with LUSC with high prognostic risk than in those with lower risk. By comparing the expression profiles, we also found significant overexpression of several immune checkpoint genes, such as *CD47* and *SIRPA* , in the high-risk group compared with the low-risk group. *CD47* is an antiphagocytic molecule, and its increased expression is significantly correlated with tumor progression and shorter survival in lung cancer. 46 Yang et al further proved our findings and suggested an increased expression of *SIRPA* in patients with LUSC at higher prognostic risks. 47 We also found DEGs between risk groups, and these DEGs were mainly enriched in the humoral immune response. These results indicated that poor prognosis in LUSC may be associated with antitumor immune dysregulation. Furthermore, ferroptosis in cancer cells can produce excess oxidized lipid mediators that affect antitumor immunity, but the ferroptosis regulation of tumor immunity is complex and reciprocal. 48 Hence, how the gene signatures identified in this study affect tumor immunity through ferroptosis warrants further investigation.

LUSC has the second highest frequency of somatic mutations, including exon mutation genome rearrangement and copy number variation, which are significantly higher than those in lung adenocarcinoma. 49 Therefore, we performed TMB analysis on samples from different risk groups, and our results suggest that patients with LUSC have a favorable overall survival benefit from TTN and TP53 mutations. Another TCGA-based study supported our findings and showed that missense mutations in TTN are independent indicators of the benign prognosis for patients, which is specific only in LUSC. 50 Additionally, tp53-derived genetic characteristics are also considered as independent prognostic predictors of patients with LUSC. 42 These mutations may also partially explain the molecular regulatory mechanism of ferroptosis in LUSC prognosis, but further experimental research is needed.

The lack of experimental exploration on how gene signatures affect tumor prognosis and immunity through ferroptosis-related regulatory mechanisms is one of the deficiencies of this study. Second, we did not correlate the prognostic risk of patients with LUSC with clinical clinicopathological features because of the lack of samples and clinical information. Despite the difficulties in collecting LUSC solid tumor samples, we will further advance this process to obtain larger sample sizes and more detailed prognostic information for subsequent mechanistic studies to explore the immune regulatory mechanisms of gene signatures on the induction of ferroptosis-related pathways in LUSC.

## Conclusions

This study identified three ferroptosis-related gene signatures ( *HELLS* , *POLR2H* , and *POLE2* ) with immune properties as potential biomarkers for LUSC prognosis. The prognostic model constructed by these three genes was an independent prognostic factor and showed potential in predicting LUSC prognostic risks and antitumor immunity. These findings provide new insights into ferroptosis-related regulation mechanisms in LUSC.
