# Supplementary material for: The Evaluation of Prognostic Value and Immune Characteristics of Ferroptosis-Related Genes in Lung Squamous Cell Carcinoma
Source: Glob Med Genet. 2023 Oct 30;10(4):285–300. doi: 10.1055/s-0043-1776386 (PMC10615648; doi:10.1055/s-0043-1776386)
Supplement: Supplementary file 1 — Supplementary Material [file 10-1055-s-0043-1776386-s2300061.pdf]

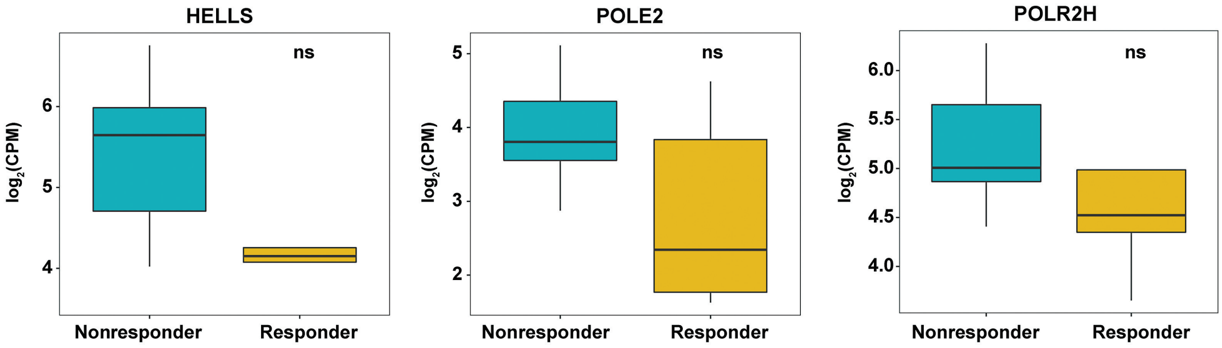

Supplementary Fig. S1 Expression differences of gene signatures in patients with NSCLC with different anti-PD-1 therapy responses.

 © 2023. The Author(s).
